# Treatment of persistent flap closure with fluid gas exchange after inverted internal limiting membrane flap technique for idiopathic macular hole

**DOI:** 10.1097/MD.0000000000035809

**Published:** 2023-11-03

**Authors:** Tsung-Tien Wu, Kai-Ling Peng

**Affiliations:** a Department of Ophthalmology, Kaohsiung Veterans General Hospital, Kaohsiung, Taiwan, R.O.C.; b School of Medicine, National Yang Ming Chiao Tung University, Taipei, Taiwan, R.O.C.

**Keywords:** flap closure, fluid gas exchange, idiopathic macular hole, inverted internal limiting membrane flap technique, vitrectomy

## Abstract

We evaluated the results of fluid-gas exchange (FGE) for long-term flap closure of idiopathic macular holes (MH) using the inverted internal limiting (ILM) flap technique. We retrospectively included eyes showing flap closure without complete MH closure and connection of separate macular tissue 1 month postoperatively after the inverted ILM flap technique was detected by ocular coherence tomography at follow-up. Eyes remained flap closure at 2 months after surgery further underwent in-office FGE with 16% C_3_F_8_. Of the 153 eyes using the inverted ILM flap technique for idiopathic MH between June 2015 and November 2018, 10 eyes (6.99%) remained flap closure at 1 month postoperatively. Among 10 eyes, 5 eyes (50%) showed flap closure at 2 months postoperatively further underwent FGE for complete MH closure, while the remaining 5 eyes (50%) progressed directly to normal macular structures at 2 months postoperatively. Improvement in vision of all flap closure from baseline was significant (*P* = .015), with a mean baseline vision of 1.19 [Snellen equivalent (SE), 20/307] ± 0.52 logMAR and the mean final vision of 0.63 (SE, 20/85) ± 0.38 logMAR. The group that underwent FGE showed better final vision of 0.45 (SE, 20/75) ± 0.23 logMAR than the group that did not undergo FGE (0.81 [SE, 20/128] ± 0.44 logMAR). All eyes achieved complete MH closure, including the eyes that underwent FGE in a mean period of 5.60 months (range 3–10 months) after the inverted ILM flap technique. Eyes that underwent FGE achieved a higher rate of foveal restoration [complete external limiting membrane 80%; complete ellipsoid zone (EZ) 60%] than those that did not receive FGE (complete external limiting membrane: 40%; complete EZ: 10%). Eyes with persistent flap closure for more than 2 months postoperatively that underwent FGE showed accelerated complete MH closure, better final vision, and foveal restoration.

## 1. Introduction

Michalewska et al first described the inverted internal limiting membrane (ILM) flap technique for an idiopathic full-thickness macular hole (MH) in 2009^.[1]^ This technique is widely used and useful in specific conditions such as large MH, myopic MH, secondary retinal detachment (RD) from MH, and uveitis.^[2–5]^ Michalewska et al introduced a modified version of the classic ILM flap technique, the temporal ILM flap technique, in which the ILM was peeled only on the temporal side of the fovea and this temporal ILM flap was inverted to cover the MH.^[6]^

Flap closure,^[6,7]^ just a thin ILM flap covering the MH, is a specific and crucial transition period for complete closure of the MH, reconnection of the two-end macular structures, and foveal restoration, especially after the inverted ILM flap technique for MH. The inverted ILM flap, which completely covers the MH, acts as a bridge between the edges of the MH to allow Müller cells migration and gliosis.^[8]^ Shiode et al further reported that Müller cell migration and gliosis are induced in a “dry” environment, where the ILM acts as a scaffold for separation from vitreous fluid in the absolute dry period after fluid-gas exchange (FGE).^[9]^

Furthermore, Boninska et al reported that flap closure occurred in 14% to 16% of cases, and even a thin ILM flap covering the MH after surgery would allow macular reconnection and foveal restoration to continue from the external limiting membrane (ELM), followed by the ellipsoid zone (EZ)^.[10]^ However, the intervals between the flap closure phases varied across previous studies, ranging from several months to more than a year, without visual improvement after the inverted ILM flap.^[6,7,11]^ No further treatment has been proposed until all layers of the macular structures completely healed themselves for vision recovery. In view of the findings of Shiode et al, our study used simple in-office FGE for long-term flap closure to complete MH closure and to reconnect the two ends of the macular structures in a prolonged dry environment.

## 2. Methods

### 2.1. Ethics statements

This study adhered to the principles of the Declaration of Helsinki and was approved by the hospital’s institutional review board (IRB). This study was approved by the Institutional Review Board of Kaohsiung Veteran General Hospital (approval number: 20-CT6-08).

### 2.2. Study design and patient selection

We retrospectively reviewed the medical records of consecutively recruited patients who underwent pars plana vitrectomy and ILM peeling with a superior inverted ILM flap covering the MHs for idiopathic MH between June 2015 and November 2018. The inclusion criteria were eyes with idiopathic MH maintained at the status of ILM flap closure 1 month after the inverted ILM flap technique. The exclusion criteria were eyes with an axial length (AL) of ≥26 mm or a refraction error > −6 diopters, RD, macular epiretinal membrane with a lamellar hole or pseudohole, MH secondary to causes such as diabetic retinopathy, trauma, or uveitis; or a history of previous vitreoretinal surgery.

Three hundred and four eyes with MHs of various causes underwent surgery by a single retinal surgeon from June 2015 to November 2018. Among them, 227 eyes underwent the inverted ILM flap technique. We further excluded eyes with high myopia (AL > 26 mm; 43 eyes) and MH secondary to causes such as diabetic retinopathy (11 eyes), branch retinal vein occlusion (3 eyes), trauma (3 eyes), rhegmatogenous RD (8 eyes), and a history of previous vitreoretinal surgery (14 eyes). Therefore, 143 eyes with idiopathic MH that underwent inverted ILM flap technique were assessed. Finally, 10 (6.99%) eyes showed ILM flap closure on the ocular coherence tomography (OCT) images 1 month after inverted ILM flap technique and were included in our study.

### 2.3. Evaluation and data collection between 2 groups

The presence of an ILM flap that covered the MH was defined as flap closure, a type of MH closure.^[6,7]^ Reconnection of the 2 ends of the macular structures was defined as complete MH closure, and complete restoration of the layers of the ELM and EZ was defined as foveal restoration. These 10 eyes were kept at the status of ILM flap closure on OCT images 1 month postoperatively and then grouped according to whether they showed complete MH closure on OCT images obtained 2 months postoperatively. Eyes with flap closure without below tissue connection at 2 months postoperatively further underwent additional in-office FGE with 16% C_3_F_8_ gas. Ten patients underwent a detailed ophthalmologic examination before and after inverted ILM flap technique as baseline and postoperative data and additional in-office FGE as pre-FGE and post-FGE information, including measurement of best-corrected visual acuity using the Snellen chart, AL or refraction measurement, slit-lamp biomicroscopy, indirect ophthalmoscopy, and fundus photography after dilation of the pupils with 10% phenylephrine and 1 % tropicamide. The microstructure of the MHs was evaluated using spectral-domain OCT (RTVue scanner; Optovue, Inc., Fremont, CA). Multiple scans were centered on the fovea, and the scan length was 6.0 or 9.0 mm. MH size was measured parallel to the retinal pigment epithelium at the closest point of the retinal apposition. The medical records contained data related to sex, age, refractive errors, lens status, size of the original MHs including minimal, and base diameters, OCT findings including central foveal thickness (CFT), MH closure type, foveal restoration, complications before and after inverted ILM flap techniques, and additional FGE in the office.

#### 2.3.1. Surgical techniques of vitrectomy and inverted ILM flap.

Standard 23-gauge pars plana vitrectomy was performed for all patients with idiopathic MH. Epiretinal membranes were peeled if present, followed by ILM peeling and tamponade with 16% perfluoropropane gas (C_3_F_8_). As brilliant blue G is not available in Taiwan, we used 0.05% indocyanine green to stain the ILM for approximately 20 to 30 seconds and informed patients about possible toxicities. With the ILM inverted flap technique, the ILM was not completely peeled, but was left in place with a superior flap around the MH and attached to the MH edges. The superior ILM flap of appropriate size to cover the MH was then inverted and gently covered with ILM forceps. All patients were instructed to maintain a face-down position for 2 weeks postoperatively. None of the study patients underwent concurrent cataract surgery using the ILM flap technique.

#### 2.3.2. FGE treatment.

In-office FGE was performed in the clinic with the patient in the lateral decubitus position with the face positioned at the lateral margin of the surgical bed with high elevation. A 30-gauge needle with a 10c.c. syringe filled with 16% C_3_F_8_ gas was inserted 3 to 4 mm posterior to the temporal limbus. In the first step, some gas is pushed into the vitreous cavity, and the level of gaseous fluid is detected through the cornea and lens. The intraocular pressure was then checked by touching the eyeball with the finger to determine whether the eyeball was too hard. Third, the syringe was pulled out to aspirate vitreous fluid. These 3 procedures were repeated to allow the eyeball full of 16% C_3_F_8_ gas. The patients maintained a face-down posture for 2 weeks after FGE in the office.

### 2.4. Statistical analysis

For data analysis, visual acuity values were converted into the logarithm of the minimum angle of resolution for calculation. Baseline and postoperative visual acuities were compared using the Wilcoxon signed-rank test. We used the Mann–Whitney U test to compare continuous variables, such as age, refraction error, visual acuity, and duration of follow-up between the groups. For categorical variables, we compared differences between groups using the chi-square test or Fisher exact test in terms of sex, lens status, and restoration of ELM and EZ. Data were analyzed using the IBM SPSS statistical software (version 20.0; IBM, Armonk, NY). Statistical significance was set *P*-value < 0.05.

## 3. Results

### 3.1. Baseline characteristics and patient demographics

Among the 143 patients with idiopathic MH, 10 eyes (6.99%) showed ILM flap closure on OCT images at 1 month postoperatively after the inverted ILM flap technique. The patients’ mean age was 60.60 ± 3.69 years. Women accounted for 70% (7/10). Among these 10 eyes, 5 (50%) eyes still showed only the ILM flap covering the MHs without complete MH closure and foveal restoration at 2 months postoperatively. In addition, they underwent FGE, while the other 5 (50%) eyes directly progressed to complete MH closure at 2 months postoperatively. Table 1 summarizes the characteristics of the overall patient population and the two flap closure groups. The general data of the two flap closure groups showed that the final visual outcomes were not significantly different with respect to sex, lens status, age, refraction error, MH size, baseline vision, complete MH closure time, and baseline and postoperative CFT. The two groups showed no significant differences in sex, lens status, age, refraction error, MH size, baseline and final visual acuity, or baseline and postoperative CFT (Table 1).

**Table 1:** General data of 2 groups of flap closure.

	Total, n = 10	*P*	Underwent FGE, n = 5	Did not undergo FGE, n = 5	*P*
Male, n (%)	3 (30)	0.833‡	1 (20)	2 (40)	.490†
OD, n (%)	6 (60)	1.000‡	4 (80)	2 (40)	.524†
Phakic, n (%)	9 (90)	1.000‡	5 (100)	4 (80)	.292†
Age (mean SD), y	60.60 (3.69)	0.149§	58.40 (3.36)	62.80 (2.68)	.056‡
Snellen equivalent (mean SD), d	0.65 (1.19)	0.778§	0.85 (0.60)	0.45 (1.64)	.841‡
Epiretinal membrane, n (%)	2 (20)	0.089‡	0 (0)	2 (40)	.114‡
Preoperative CFT (mean SD), μm	408.60 (56.03)	0.210§	412.00 (64.32)	405.20 (53.82)	.861‡
*Size of original MH*					
Minimal diameter (mean SD), μm	250.89 (67.99)	0.620§	243.32 (52.53)	258.46 (86.59)	1.000‡
Base diameter (mean SD), μm	495.34 (179.55)	0.842§	504.07 (237.95)	486.62 (125.39)	.841‡
Baseline BCVA, logMAR (mean SD)	1.19 (0.52)	0.709§	1.06 (0.57)	1.32 (0.48)	.310‡
Final BCVA, logMAR (mean SD)	0.63 (0.38)		0.45 (0.23)	0.81 (0.44)	.222‡
MH closure time (mean SD), months	3.60 (3.20)	0.052§	5.60 (3.58)	1.60 (0.55)	.007‡^,^*
Final CFT, (mean SD), μm	246.20 (32.88)	0.920§	260.40 (27.19)	232.00 (34.48)	.222‡
Follow-up time (mean SD), months	14.30 (9.01)	0.017§^,^*	14.40 (4.83)	14.20 (12.64)	.690‡

BCVA = best-corrected visual acuity; CFT = central foveal thickness; d = days; FGE = fluid gas exchange; logMAR = logarithm of minimum angle of resolution; MH = macular hole; n = number; SD = standard deviation; y = years; % = percentage.

† Chi-square or fisher exact test.

‡ Mann–Whitney U test.

§ Pearson correlation.

* *P* < .05.

### 3.2. Functional outcomes

The mean final best-corrected visual acuity in the group that received additional FGE was 0.45 [Snellen equivalent (SE), 20/75] ± 0.23 logMAR and that in the group without FGE was 0.81 (SE, 20/238) ± 0.44 logMAR. Although the final vision was not significantly different between the 2 groups, the mean final vision in the FGE group was superior to that in the non-FGE group. Follow-up evaluations revealed no complications in any of the 10 patients.

**Figure 1.**
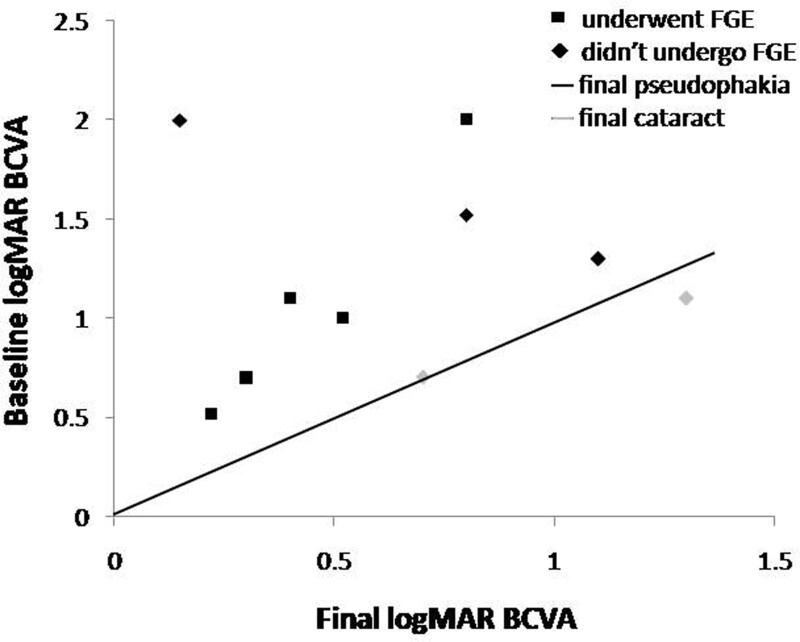
Distribution of baseline and final logMAR BCVA of all patients. The slant line indicates that the patient’s baseline and final logMAR BCVA were equal. The spots in the left zone of the slant line indicate that the patient had poor baseline logMAR BCVA but showed better final logMAR BCVA. A total of 8 patients showed spots in the left zone of the slant line. One patient who showed a spot on the slant line and a patient who showed a post in the right zone of the slant line had persistent cataract at the final visits.

Figure 1 shows that 8 (80%) patients had better final vision than baseline vision. All eyes that underwent FGE showed better final vision than baseline vision. However, 2 eyes (20%) that did not undergo FGE showed similar or worse final vision, and their cataracts persisted at the final visit. A patient had a baseline vision of 0.7 logMAR (SE, 4/20) and showed the best vision at 1 month postoperatively (0.52 logMAR; SE, 6/20), which was much better than the final vision of 0.7 logMAR (SE, 4/20). The other patient had a baseline vision of 1.1 logMAR (SE, 16/200) and showed the best vision at 1 month postoperatively (1.1 logMAR; SE, 16/200), which was relatively better than the final vision of 1.3 logMAR (SE, 10/200). Vision in these 2 eyes may improve further after cataract surgery.

Table 2 presents the baseline and postoperative vision data for all patients and the different groups. Significant improvements in final vision were observed from baseline in the flap closure (*P* = .013) and FGE groups (*P* = .043). For total 10 eyes, the mean postoperative vision gradually improved from 1.19 (SE, 20/307) ± 0.52 logMAR at baseline to 0.92 (SE, 0.92) ± 0.21 logMAR at 3 months postoperatively and mildly worsened to 0.96 (SE, 20/181) ± 0.28 logMAR at 12 months postoperatively, but obviously improved to 0.63 (SE, 20/85) ± 0.38 logMAR at the final visit. The mean postoperative vision in the FGE group slowly improved from 1.06 (SE, 20/230) ± 0.57 logMAR at baseline to 0.90 (SE, 20/158) ± 0.57 logMAR at 3 months postoperatively, and gradually improved to 0.80 (SE, 20/125) ± 0.14 logMAR at 12 months postoperatively but improved to 0.45 (SE, 20/56) ± 0.23 logMAR at the final follow-up. However, the mean postoperative vision in the group without FGE gradually improved from 1.32 (SE 20/414) ± 0.48 logMAR at baseline to 0.94 (SE, 20/174) ± 0.18 logMAR at 3 months postoperatively. It then gradually worsened to 1.30 (SE, 20/395) ± 0.00 logMAR at 12 months postoperatively, but subsequently improved to 0.81 (SE, 20/128) ± 0.44 logMAR at final visit. The difference in vision between the 2 groups was significant at 12 months postoperatively (*P* = .009), with 4 patients (80%) in the group that did not receive FGE still showing cataract and had poor vision at 6 and 12 months postoperatively.

**Table 2:** The preoperative and postoperative visions of total patients and 2 groups.

BCVA logMAR	Total (mean SD)/P SE	Underwent FGE (mean SD)/P SE	Did not undergo FGE (mean SD)/P SE	*P*
Pre-operation	1.19 (0.52) 20/307	1.06 (0.57) 20/230	1.32 (0.48) 20/414	.310†
Postoperative 1 months	1.00 (0.35)/0.173‡ 20/200	0.88 (0.31)/0.655‡ 20/150	1.15 (0.38)/0.109‡ 20/280	.310†
Postoperative 3 months (post-FGE 1 months)	0.92 (0.21)/0.176‡ 20/165	0.90 (0.57)/0.715‡ 20/158	0.94 (0.18)/0.109‡ 20/174	.690†
Postoperative 6 months (post-FGE 4 months)	0.96 (0.23)/0.237‡ 20/181	0.86 (0.13)/0.715‡ 20/143	1.08 (0.29)/0.285‡ 20/238	.286†
Postoperative 12 months (post-FGE 10 months)	0.96 (0.28)/0.138‡ 20/181	0.80 (0.14)/0.593‡ 20/125	1.30 (0.00)/0.180‡ 20/395	.009b†
Final	0.63 (0.38)/0.013‡^,^* 20/85	0.45 (0.23)/0.043‡^,^* 20/56	0.81 (0.44)/0.197‡ 20/128	.222†

BCVA: best-corrected visual acuity; FGE: fluid gas exchange; logMAR, logarithm of minimum angle of resolution; SD, standard deviation; SE, Snellen equivalent.

† Mann–Whitney *U* test.

‡ Wilcoxon signed rank test.

* *P*<.05.

### 3.3. Anatomical outcomes

The time to complete MH closure was significantly different between the two groups (*P* = .007). In both groups, all MHs eventually reconnected and closed. The mean time to complete MH closure in the FGE group was 5.60 ± 3.58 months (all listed: 3, 3, 3, 9, 10 months), and that in the group that did not undergo FGE was 1.60 ± 0.55 months (range, 1–2 months) after the inverted ILM flap technique. Two months postoperatively, the overall rate of MH closure in the eyes showing flap closure was 50% (5/10). For the group that underwent FGE, the mean interval for complete MH closure after FGE was 3.60 ± 3.58 months (all listed: 1, 1, 1, 7, 8 months) and complete MH closure occurred 1 month after FGE was 60% (3/5).

**Figure 2.**
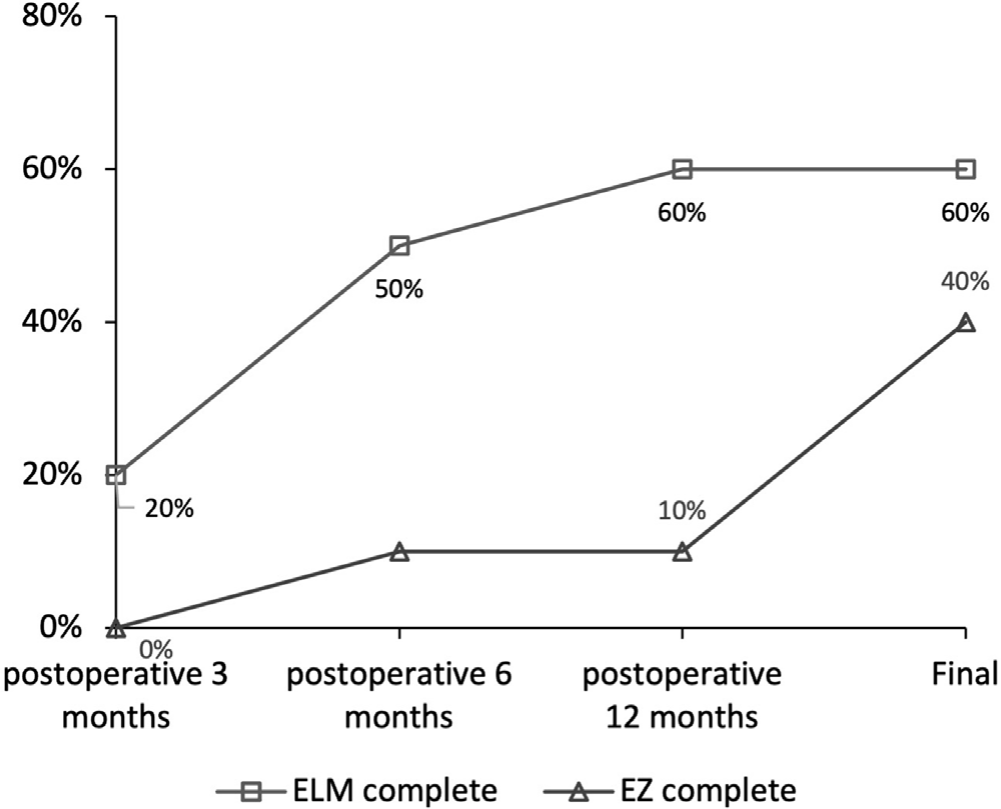
Complete ELM and EZ achieved at different time points in 10 eyes showing flap closure at 1 month postoperatively. Complete ELM was achieved in 50% of the cases at 6 months postoperatively, 60 % of the cases at 12 months postoperatively and at the final follow-up. However, complete EZ restoration was achieved in 10% of the cases at 6 months postoperatively and 40% of the cases at the final follow-up.

Figure 2 presents the findings for the presence of complete ELM and EZ at different time points in the 10 eyes with flap closure at 1 month postoperatively. Complete ELM was observed in 50% of the eyes at 6 months postoperatively, 60 % at 12 months postoperatively and 60% at the mean follow-up period of 14.30 ± 9.01 months. However, complete EZ was observed in 10% of the cases at 6 and 12 months postoperatively and 40% of the cases at the final visit with a mean follow-up period of 14.30 ± 9.01 months.

Table 3 summarizes the data on the presence of the complete ELM and EZ in the different flap closure groups. Although the two groups showed no significant differences in the presence of complete ELM and EZ, the group that received FGE showed a relatively higher rate of 80% (4/5) for a complete ELM and 60% (3/5) for a complete EZ that did not receive FGE (complete ELM, 40% [2/5]; complete EZ, 10% [1/5]) over a mean follow-up period of 14.30 ± 9.01 months.

**Table 3:** The eyes with complete ELM and EZ in 2 groups of flap closure.

	Total n = 10	Underwent FGE n = 5	Did not undergo FGE, n = 5	*P*
ELM complete, n (%)	6 (60)	4 (80)	2 (40)	.197
EZ complete, n (%)	4 (40)	3 (60)	1 (10)	.197

ELM = external limiting membrane, EZ = ellipsoid zone; FGE: fluid gas exchange.

*P<0.05, Chi-square or Fisher exact test.

**Figure 3.**
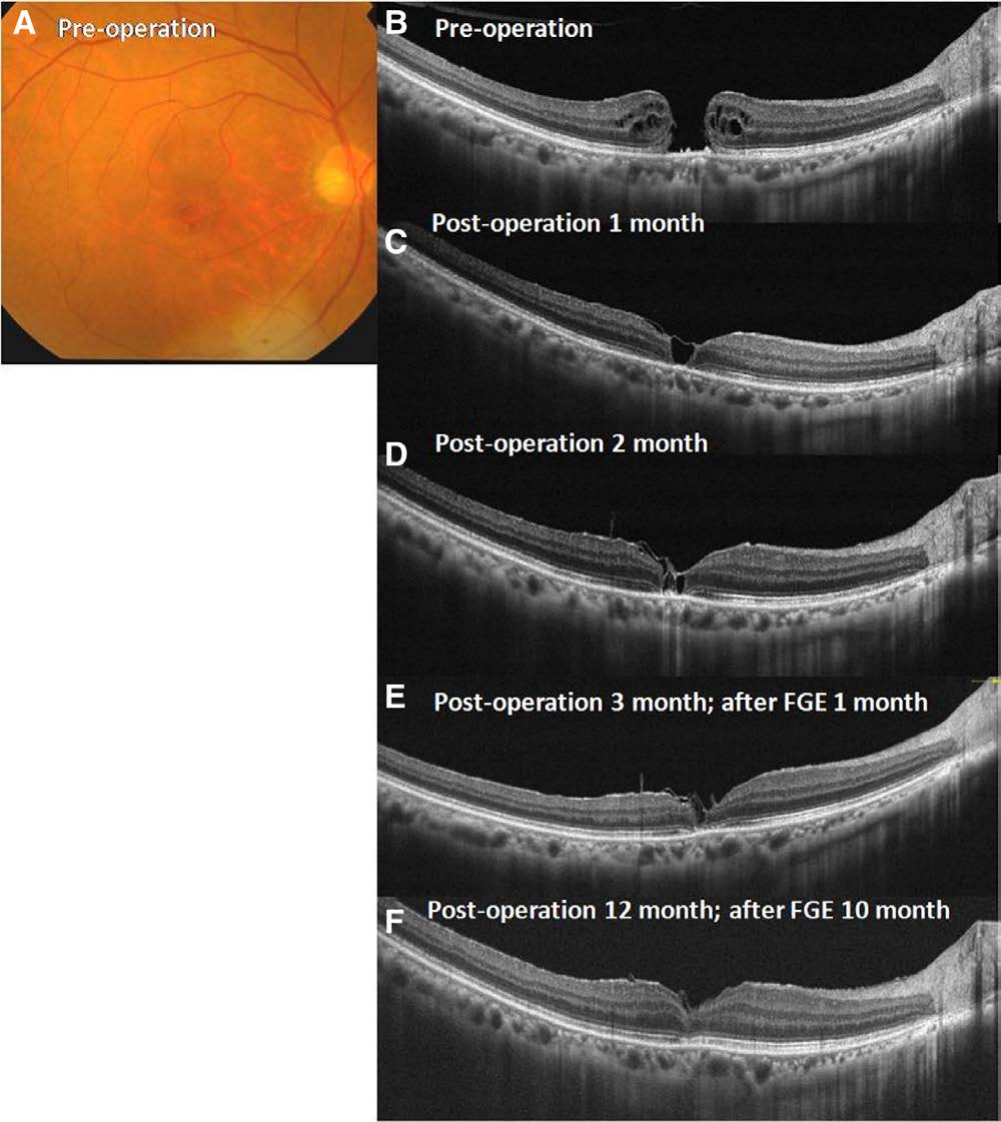
A 54-year-old female patient presented with idiopathic MH on fundus photography and OCT (A and B). A thin ILM flap covered the MH from fundus images and OCT images obtained 1 and 2 months postoperatively (C and D). The patient then underwent further FGE. A thin ILM flap with reconnection of the apart macular tissue was found 3 months postoperatively (E). Horizontal views of OCT images showed a good foveal contour and complete ELM and EZ with CFT of 271 μm at 12 months postoperatively (F). CFT = central foveal thickness; ELM = external limiting membrane; EZ = ellipsoid zone; FGE = fluid-gas exchange; ILM = inverted internal limiting membrane; MH = macular hole; OCT = optical coherence tomography.

Figure 3 shows the findings of a 54-year-old female patient who showed ILM flap closure at 1 month (Fig. 3C) and 2 months (Fig.3D) postoperatively in her right eye after the inverted ILM flap technique for idiopathic MH with a vision of 4/20 (Fig.3A and B). The minimum diameter of the MH was 166.67 μm while the base diameter was 305.56 μm. Without complete MH closure and foveal restoration, her right eye underwent further FGE two months postoperatively. The inner retina was reconnected between the edges of the MH at 3 months postoperatively (Fig. 3E). The patient underwent cataract surgery in the right eye for 9 months postoperatively. Twelve months postoperatively, complete ELM and EZ with a CFT of 271 μm were observed on horizontal views of the OCT images and her vision improved to 8/20 (Fig. 3F). Her final vision improved to 10/20 at 24 months postoperatively.

## 4. Discussion

Casini et al compared the classic inverted ILM technique proposed by Michalewska et al and the modified method described by Casini et al and found that the rates of flap closure at 3 months postoperatively were 7.5% and 10.26%, respectively, for large MH^[7]^ but both methods did not yield significantly different flap closures at 6 months postoperatively. In a study by Michalewska et al, the rate of flap closure at 6 months postoperatively after the temporal inverted ILM flap technique was 6%, and the rate 12 months postoperatively after the classic inverted ILM flap technique was 3%^6^ with flap closure maintained without complete MH closure. In the study by Tsui et al, the incidence of flap closure after temporal flap of the inverted ILM flap technique for non-high myopia group of MH was 31.25% (5/16), higher than previous studies, and all cases in the flap closure status remained within 4 months.^[11]^ In our study, 6.99% of the eyes showed ILM flap closure 1 month postoperatively after the superior inverted ILM flap technique for idiopathic MHs. However, all flap closures, including those performed using in-office FGE, progressed to complete MH closure within 12 months postoperatively. Furthermore, the mean time required to connect the 2 ends of the macular structures and achieve complete MH closure was 3.60 months (range, 1–8 months) after FGE in the FGE group. Thus, all eyes in the FGE group that underwent FGE required a mean time of 5.60 months (range, 3–10 months) after the inverted ILM flap technique to achieve complete MH closure, slightly longer than 4 months but <12 months, as reported by Tsui et al with the temporal flap of the inverted ILM flap technique and Michalewska et al with the classic inverted ILM flap technique. In fact, 60% (3/5) of the eyes at the flap closure status performed in the office FGE progressed to complete MH closure 1 month after FGE, 3 months after the inverted ILM flap technique. Therefore, the use of in-office FGE in our study accelerated the connection between the two ends of the macular tissue and shortened the flap closure interval.

The percentage of ILM flap closure in our study was less than that reported by Casini et al and Michalewska et al, while the mean minimum diameter and base diameter of MH in our study were 250.89/ 495.34 (36.00/179.57) μm, which is smaller than those reported by Casini et al^[7]^ (561/836 and 603/884 μm), Michalewska et al^[6]^ (544/894 μm), and Tsui et al^[11]^ (non-high myopia group, 738/1582 μm). MH with a larger minimal diameter may show a higher rate of flap closure after inverted ILM flap techniques. However, in these studies, the MH shape showed exaggerated differences between the minimum and the base diameters.

Faria et al proposed that one of the following situations may occur after inverted ILM flap techniques: (1) The ILM flap flips back, resulting in surgical failure; (2) the ILM flap dips into the hole, which contacts the inner lining of the hole; and (3) the ILM flap remains over the hole in the intended position. They further suggested that the third situation is ideal because the hole will close; with better visual outcomes and outer retinal layer realignment.^[12]^ Some studies have reported that the final visual acuity is lower in eyes with flap closure than in eyes that initially develop complete MH closure,^[10]^ including U-, V-type, or irregular closure.^[13,14]^ However, all of the cases in our study showed the third situation at 1 month postoperatively. All patients showed complete MH closure and foveal restoration on OCT during follow-up evaluations. Although vision was not significantly different between the preoperative and 1-, 3-, 6- and 12-postoperative intervals overall or in each group, it was significantly different between the groups with and without FGE at 12 months postoperatively (*P* = .009) because more patients who did not receive FGE also did not undergo cataract surgeries. Additionally, the baseline and final vision in eyes with flap closure were significantly different (*P* = .013), particularly in eyes with FGE (*P* = .043).

Regarding foveal restoration after the temporal inverted ILM flap technique, Michalewska et al reported that at 12 months postoperatively, ELM defects were still present in 25% of the cases, and EZ defects were present in 57% of the cases.^[6]^ Casini et al compared the classic and modified types of inverted ILM flap techniques and reported ELM defect rates of 20%/ 28.21% and EZ defect rates of 45% and 51.28% at 12 months postoperatively.^[7]^ Boninska et al compared flap closure with an initially developed MH closure and reported that ELM and EZ reconnection did not differ between the two groups, whereas reconstruction of the ELM preceded restoration of the EZ.^[10,15]^ However, for all flap closures in our study, 60% patients showed complete ELM and 10% showed complete EZ at 12 months postoperatively while 60% showed complete ELM and 40% showed complete EZ at mean follow-up of 14.30 ± 9.07 months, which were relatively slower than the foveal restoration rates reported in previous studies using different methods of inverted ILM flap technique, not specifically for the flap closure. Eyes that underwent FGE showed late-onset complete MH closure, whereas those that did not undergo FGE showed early complete MH closure. For foveal restoration, we further found that eyes that underwent FGE showed higher completion rates of ELM (80%) and EZ (60%) than those who did not undergo FGE at mean follow-up period of 14.30 ± 9.01 months. The completion rates of ELM and EZ in the eyes that underwent FGE approached those reported in studies using different methods of the inverted ILM flap technique, not specifically for flap closure. Furthermore, the group that underwent FGE showed a late onset of complete MH closure but better final vision because of a higher rate of foveal restoration. Additional in-office FGE created a prolonged period of dry space for long-term flap closure to accelerate Müller cell migration and gliosis along the inverted ILM flap and to reconnect the entire layers of both ends of the separated macular structures, including enhancing foveal restoration of the ELM and EZ.

This study had some limitations, including its retrospective design, small sample size, variability and short follow-up period in the group that did not undergo FGE.

## 5. Conclusion

In conclusion, we performed additional in-office FGE for eyes that were still in the flap closure stage two months postoperatively. Both flap closure groups achieved complete MH closure within 10 months of the surgery. Although the eyes that received FGE showed a later onset of complete MH closure, the final vision in these eyes was significantly better and foveal restoration was higher. Thus, the visual and anatomical outcomes of FGE for eyes with persistent flap closure more than 2 months postoperatively are not inferior to those without FGE and progress directly to normal macular structures more than 2 months postoperatively.

## Author contributions

**Conceptualization:** Tsung-Tien Wu.

**Formal analysis:** Kai-Ling Peng.

**Investigation:** Kai-Ling Peng.

**Methodology:** Tsung-Tien Wu.

**Supervision:** Tsung-Tien Wu.

**Writing – original draft:** Kai-Ling Peng.

**Writing – review & editing:** Tsung-Tien Wu.
